# The Challenge of Growth Hormone Deficiency Diagnosis and Treatment during the Transition from Puberty into Adulthood

**DOI:** 10.3389/fendo.2013.00034

**Published:** 2013-03-20

**Authors:** Elena Inzaghi, Stefano Cianfarani

**Affiliations:** ^1^Molecular Endocrinology Unit, Bambino Gesù Children’s HospitalRome, Italy; ^2^Department of Women’s and Children’s Health, Karolinska InstitutetStockholm, Sweden

**Keywords:** GH, transition to adult care, IGF-I, pituitary gland, GH therapy

## Abstract

In children with childhood-onset growth hormone deficiency, replacement GH therapy is effective in normalizing height during childhood and achieving adult height within the genetic target range. GH has further beneficial effects on body composition and metabolism through adult life. The transition phase, defined as the period from mid to late teens until 6–7 years after the achievement of final height, represents a crucial time for reassessing children’s GH secretion and deciding whether GH therapy should be continued throughout life. Evidence-based guidelines for diagnosis and treatment of growth hormone deficient children during transition are lacking. The aim of this review is to critically review the up-to-date evidence on the best management of transition patients in order to ensure the correct definitive diagnosis and establish the appropriate therapeutic regimen.

## Introduction

The main effect of GH therapy in childhood is to stimulate linear growth: therefore the primary objective of GH replacement therapy in growth hormone deficient (GHD) children is to achieve adult height within the genetic target range. In addition, GH plays a key role in the regulation of body composition and metabolism and its deficiency in adulthood has been associated with reduced lean body mass and bone mineral density (BMD), increased visceral adiposity, abnormal lipid profile, decreased muscle strength, cardiovascular risk, and impaired quality of life (Rosén and Bengtsson, 1990; Cuneo et al., 1992, 1993; Rosén et al., 1993; de Boer et al., 1994, 1995; Weaver et al., 1995; Attanasio et al., 1997; Johansson et al., 2004). These non-growth promoting effects of GH are considered so important for body homeostasis to require lifelong GH administration in subjects with permanent GHD (Molitch et al., 2006). As many subjects are no longer GH-deficient when retested at the end of linear growth, the correct reassessment of childhood-onset GHD (CO-GHD) is crucial for selecting patients who need lifelong GH therapy.

Transition phase has been defined as the period of life starting in late puberty and ending with full adult maturation (i.e., from mid to late teenage years until 6–7 years after achievement of final height) (Gordon et al., 1991; Matkovic et al., 1994; Clayton et al., 2005).

During transition GH is effective in maintaining body proportions and metabolic balance. In addition, significant psychosocial adjustment takes place during this time frame. Therefore, CO-GHD patients should not be declared adults as soon as they achieve adult height, but should receive specific care in the context of a transition program managed by pediatric or adult endocrinologists experienced in the management of hypopituitarism and GHD.

## Peculiarities of GH Deficiency from Childhood to Adulthood

Features of adult GHD syndrome are summarized in Table 1.

**Table 1:** **Features of growth hormone deficiency (GHD) in adult patients**.

**Features of GHD adult patients**
Reduced lean body mass and increased visceral adiposity
Reduced bone mineral density with increased risk of fractures
Reduced IGF-I levels
Decreased muscle strength and exercise capacity
Diminished quality of life (less cognitive function, decreased of well-being)
Abnormal serum lipid profile (increased total cholesterol, LDL cholesterol, triglycerides, lipoprotein A, apolipoprotein B; decreased HDL cholesterol)
Lower cardiac function, impaired left ventricular performance, and increased prevalence of cardiovascular disease

It has been reported that GH replacement therapy in adulthood normalizes metabolism and body composition (Molitch et al., 2006), and may improve the quality of life of GHD patients (McGauley, 1989).

Transition years represent an important phase of growth process when somatic development reaches its completion. Several studies have tried to evaluate the consequences of either withdrawing or continuing GH therapy in transition subjects, often arriving at conflicting results in terms of both magnitude of response and dose-effect relationship (Table 2).

**Table 2:** **Alterations during transition phase in CO-GHD patients**.

Author	*N* (age, years)	Body composition	IGF-I	BMD	Muscle	QoL	Serum lipids	Carbohydrate metabolism	Other parameters
Johannsson et al. (1999)	56 (16–20)	↓ LBM and ↑ BF both in severe GH deficiency patients and in those having sufficient endogenous GH secretion (more marked than in controls), ↑ of truncal fat in GHD subjects	↓IGF1 after 2 year off-therapy in subjects with severe GHD	NA	NA	NA	↓ HDL-c and ↓ TG in GHD group	↓ Serum insulin both in severe GH deficiency patients and in those having sufficient endogenous GH secretion	No changes in FT4 and FT3 concentrations ↓ SBP in patients having sufficient GH secretion
Nørrelund et al. (2000)	18 (20 ± 1)	↑ TBF in placebo group; ↑ FFM when GH therapy is restarted in placebo group	NA	NA	NA	NA	↓ Rates of lipid oxidation in placebo group	↑ IS in placebo group; ↓ IS when GH therapy is restarted	
Vahl et al. (2000)	19 (20.2 ± 0.65)	TBF and WC ↑ in placebo group	↓ In placebo group	NA	No significant changes	Placebo group have lower total score	↑ HDL-c in the GH group	↓ Fasting glucose in the placebo group	↓ Of FT3 in placebo group
Lanes et al. (2001)	19 (Ut: 14.2 ± 2.8; T: 14.4 ± 2.6)	NA	NA	NA	NA	NA	LDL-C and lipoprotein(a) > in untreated patients compared to controls	NA	No differences in mass and cardiac function between groups
Colao et al. (2002)	20 (17–20)	NA	↓ in GHD subjects after 6 months off-therapy	NA	NA	NA	↑ TG, ↑ LDL, ↑ total/HDL cholesterol ratio in GHD patients after stop therapy	No changes	↓ LVM, ↑ fibrinogen levels,↓ E/A in GHD subjects off-therapy
Attanasio et al. (2002)	127 (CO: 20.9 ± 2.4; AO: 25.2 ± 3)	CO-GHD patients have <BMI, <LBM, <FM than AO-GHD	IGF1 is < in CO-GHD than AO-GHD	CO-GHD show < BMC	NA	NA	NA	NA	
Drake et al. (2003)	24 (17 ± 1.4)	NA	NA	↑ BMC and mean lumbar BMD in ongoing GH subjects	NA	NA	NA	NA	
Shalet et al. (2003)	149 (AD: 19.4 ± 2.7; PD: 19.6 ± 2.8)	NA	A dose-dependent ↑	Total BMC ↑ in treated groups, predominantly at the lumbar spine	NA	NA	NA	NA	↑ Bone-specific alkaline phosphatase in GH-treated group
Underwood et al. (2003)	64 (23.8 ± 4.2)	↑ FM in placebo group; ↓ FM in GH-treated group	↑ In high GH dose treated group-dose	Dose-related ↑ spine BMD	NA	No significant effects among groups	↓ LDL in high GH dose treated group	NA	No changes in cardiac structure or function
Attanasio et al. (2004)	139 (AD: 19.4 ± 2.7; PD: 19.6 ± 2.8)	↑ LBM in GH-treated patients, ↓ fat mass	No differences among groups	NA	NA	NA	↑ Total C and LDL/HDL ratio in non GH-treated patients; ↓ LDL/HDL-C in pediatric dose subjects	NA	
Carroll et al. (2004)	24 (17 ± 0.3)	↑ LBM in GH-treated patients	↓ IGF1 in patients off-therapy	NA	NA	NA	No differences between two groups	↑ Is after GH cessation	
Mauras et al. (2005)	58 (15.8 ± 1.8)	No differences between groups	Less reduction in GH-treated group (no significative)	No differences between groups	No differences between groups	No differences between groups	No differences between groups	No differences between groups	
Koltowska-Häggström et al. (2010)	313 (IGHD: 17.5 ± 1.84; NON-IGHD: 17.1 ± 1.91)	NA	↑ After 1 year of GH treatment	NA	NA	A longer GH gap is associated with a poorer QoL	A longer duration of GH interruption was associated with a worse lipid profile in non-IGHD patients	NA	
Bazarra-Castro et al. (2012)	75 (<25)	↑ BMI during GH therapy pause	NA	NA	NA	NA	NA	NA	

*LBM, lean body mass; BF, body fat; TG, triglycerides; SBP, systolic blood pressure; TBF, total body fat; FFM, fat-free mass; IS, insulin sensitivity; WC, waist circumference; LVM, left ventricular mass; E/A, early-to-late mitral flow velocity ratio; BMI, body mass index; LBM, lean body mass; FM, fat mass; BMC, bone mineral content; BMD, bone mineral density; QoL, quality of life; Ut, untreated group; T, treated group; CO, childhood-onset GHD; AO, adult onset GHD; AD, adult dose; PD, pediatric dose; IGHD, idiopathic GHD; NON-IGHD, non-idiopathic GHD*.

In most studies, GH treatment in the transition phase increases lean body mass and reduces fat mass, especially in males, with still conflicting data regarding the dose-response (Nørrelund et al., 2000; Vahl et al., 2000; Underwood et al., 2003; Attanasio et al., 2004; Carroll et al., 2004). However, similar effects are observed when GH replacement is resumed after a variable off-therapy time. These results have been questioned by Mauras’ study on a population of 58 CO-GHD adolescents who were randomized to receive either GH or placebo for 2 years (Mauras et al., 2005). No differences in body composition, lipid and carbohydrate metabolism, BMD, cardiac function, muscle strength, or quality-of-life were observed. However, the fact that this study comprised a higher percentage of patients with idiopathic/isolated GHD than previous studies, may account for the lack of metabolic effects induced by GH therapy. GH deficiency during the transition phase may reduce BMD thus increasing the risk of osteoporosis and fractures (Matkovic et al., 1994; Saggese et al., 1996; Johannsson et al., 1999; Attanasio et al., 2002; Drake et al., 2003; Shalet et al., 2003; Underwood et al., 2003). Therefore, Shalet (2006) proposed to continue GH treatment, without any withdrawal, to allow the attainment of peak bone mass. On the contrary, the study by Mauras et al. (2005) has shown no benefit of GH therapy continuation on BMD. Högler and Shaw (2010) support the idea that isolated CO-GHD is not associated with the risk of fractures and low bone density and that routine DXA measurements should not be recommended for children or young adults with isolated CO-GHD.

There is moderate evidence that insulin sensitivity increases after discontinuation of GH therapy, whereas no change or only a moderate increase of fasting insulin values is observed in subjects who continue therapy (Nørrelund et al., 2000; Underwood et al., 2003; Carroll et al., 2004). However, Mauras et al. (2005) found no changes in glucose metabolism related parameters in GH-treated vs. placebo-treated and control subjects.

GH treatment seems to improve the lipid profile of GHD patients (Vahl et al., 2000). A deterioration of lipid status (i.e., increased LDL and reduced HDL cholesterol levels) has been reported in GHD adolescents after discontinuation (Johannsson et al., 1999; Colao et al., 2002; Attanasio et al., 2004). Koltowska-Häggström et al. (2010) suggest that a longer GH off-therapy period is associated with a worse lipid profile, proposing that the sooner GH treatment is resumed, the better is the metabolic outcome. On the contrary, Carroll et al. (2004) showed no changes in lipid profile after discontinuation of GH.

In conclusion, there are conflicting data on the necessity to continue GH therapy during transition without interruption. The majority of studies suggest that the continuation of GH treatment would prevent the onset of metabolic alterations and deterioration of body composition, whereas the impact of GH treatment on quality of life and psychological well-being remains to be established.

## Retesting GH Secretion: Lights and Shadows

More than two thirds of children diagnosed as GH insufficient show normal GH response when retested at the end of growth (Tauber et al., 1997; Maghnie et al., 1999; Attanasio et al., 2002). In particular, there is a good chance of recovering a normal GH secretion for patients with idiopathic GHD as well as for subjects with isolated or partial GHD (Tauber et al., 1997; Maghnie et al., 1999).

Patients with known mutations or irreversible structural lesions with multiple pituitary hormone deficits (MPHD) are likely to have permanent GHD, though there may be subjects with MPHD with normal GH response at retesting (de Boer and van der Veen, 1997; Tauber et al., 1997; Shalet et al., 1998; Maghnie et al., 1999). Normalization of GH secretion is highly unlikely in patients who underwent surgery and/or cranial irradiation and never occurs in patients operated for craniopharyngioma (Tauber et al., 1997; Maghnie et al., 1999; Leger et al., 2005).

The possible causes of recovering a normal GH response to stimulation tests are summarized in Table 3 (Cacciari et al., 1992, 1994; Aimaretti et al., 2000; Maghnie et al., 2001, 2002; Radetti et al., 2007).

**Table 3:** **Possible causes of recovering a normal GH response to stimulation tests**.

**Possible causes of GH response recovering to stimulation tests at the end of linear growth**
Transient GH deficiency
Changes in diagnostic criteria or lack of reproducibility in GH stimulation testing
False positive response at the time of diagnosis in children with short stature or pubertal delay
Neurosecretory dysfunction (characterized by a normal response to provocative tests and reduced spontaneous release)
Improvement in hypothalamic-pituitary function after puberty
Different response to stimulation tests due to:
Type of stimulation test
Age
BMI
Disease duration
Number of pituitary hormone deficiencies
Pituitary abnormalities

In CO-GHD teenagers, pituitary function is re-evaluated at the end of linear growth, as defined by growth velocity of less than 1.5–2 cm/year or a bone age of at least 14.5 years in females and at least 16.5 years in males (Clayton et al., 2005; Attanasio and Shalet, 2007). It is widely accepted the necessity of a GH wash out period prior to retesting, to avoid false positive results. Nevertheless, the shortest wash out time to get reliable retesting data is still undefined. An interval of 1–3 months was considered acceptable by the GH Research Society (Growth Hormone Research Society, 2000; Geffner, 2003; Molitch et al., 2006).

**Figure 1:**
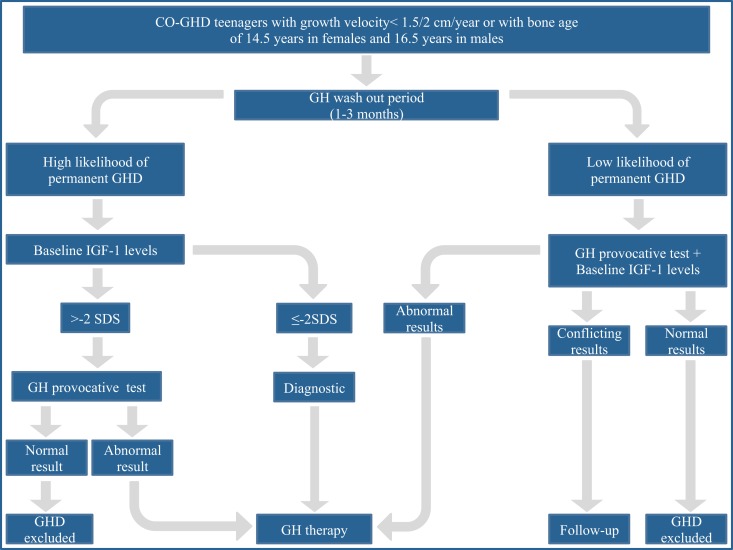
**The proposed workup for assessing transition patients with growth hormone deficiency**. From a consensus statement issued by the European Society for Pediatric Endocrinology (Clayton et al., 2005).

A proposed workup of CO-GHD patients at the end of growth is summarized in Figure 1 (Clayton et al., 2005). In brief, a single measurement of baseline IGF-I levels in patients with high likelihood of permanent GHD as a consequence of genetic causes, structural hypothalamic-pituitary anomalies, acquired hypothalamic-pituitary disease, and irradiation of the hypothalamus-pituitary area could be adequate to establish the final diagnosis. IGF-I values less than −2 SDS indicate persistent GHD, whereas values higher than −2 SDS, should prompt to perform a GH provocative test, a subnormal response confirming GHD diagnosis. In patients with low likelihood of permanent GHD, such as those with isolated idiopathic GHD, both GH provocative test and IGF-I assessment should be performed to achieve the final diagnosis. If both parameters are normal the condition of GHD can be excluded. If both parameters are low, GHD diagnosis is confirmed. Finally, in case of conflicting results re-evaluation of the case is required.

## Testing the Tests

Since it is well known that GH is secreted by the pituitary gland in a pulsatile pattern, provocative tests are needed to investigate pituitary GH secretion. There are many different GH pharmacological stimulation tests, each of them showing both advantages and limitations. The choice of the suitable GH provocative test is mainly based on the balance between reliability and safety. Although, an optimal GH stimulation test to be used in transition has yet to be established, the insulin tolerance test (ITT) has been suggested to have the best efficacy/safety ratio if performed in experienced endocrine units. ITT is considered as the “gold standard” for GHD diagnosis in adults, and allows the assessment of both GH secretion and the hypothalamus-pituitary-adrenal axis function (Geffner, 2003; Styne, 2003). However, ITT may provoke severe hypoglycemia and is contraindicated in patients on anticonvulsant drugs, with coronary heart disease or with adrenal insufficiency (Clayton et al., 2005; Molitch et al., 2006).

An alternative test is the combined stimulation with Growth Hormone Releasing Hormone and arginine (GHRH + Arg). It shows excellent sensitivity and specificity both in childhood and in adulthood, assuming appropriate cut-off limits (Corneli et al., 2007; Giacomozzi et al., 2012) but its reliability is uncertain in patients with hypothalamic impairment. Therefore, a normal GH response to GHRH + Arg test in a patient with ascertained hypothalamic alterations should induce suspicion for a false negative result. In case of discrepancy between the response to whatever GH stimulation test and IGF-I concentrations, the diagnosis should be critically reconsidered.

Glucagon, arginine, GHRH, and clonidine provocative tests have been reported to be less accurate in diagnosing GHD in transition, in that no cut-off limits have been established (Styne, 2003; Clayton et al., 2005; Gasco et al., 2008).

Although ITT and GHRH + Arg tests have been partly validated in transition, the available data are scarce and, at the moment, their use is not driven by a firmly established evidence-based approach. There is still controversy about the cut-off values to be used to discriminate between normal and abnormal GH response in patients during the transition phase. The ITT cut-off value used in adulthood, i.e., a GH peak less than 3 ng/ml, seems to be too restrictive as the highest GH response to provocative test occurs in late puberty (Clayton et al., 2005).

Bonfig et al. (2008) have reported the highest accuracy of ITT with a cut-off of 5 ng/ml, whereas Secco et al. (2009) have suggested a GH peak of 5.62 ng/ml as the best discriminator in patients with high likelihood for permanent GHD. Maghnie et al. (2005) have reported that a GH peak of 6.1 ng/ml has a sensitivity of 96% and a specificity of 100%, concluding that a cut-off of less than 5 ng/ml is too restrictive for the diagnosis of permanent GHD in the transition phase, being burdened by a high rate of false negative results.

The cut-off values in GHRH + Arg test are even more debated. The same value used in adults, i.e., GH peak of 9 ng/ml, was initially proposed. More recently, the same GH peak value used in childhood, i.e., 19 ng/ml, has been reported to achieve 100% sensitivity and 97% specificity. However, this cut-off value has been validated in a relatively small cohort of patients. Moreover, this reference was obtained in lean subjects and needs to be validated in overweight and obese patients. Obesity is associated with both decreased basal and pulsatile release of GH and lower GH response to provocative tests. Therefore, obesity related parameters, such as waist circumference, trunk fat, and abdominal visceral adipose tissue, should be considered in order to establish appropriate cut-off values (Makimura et al., 2008).

In conclusion, many factors can influence the definition of cut-off limits in transition, such as the different etiology of GHD and the age at retesting, yielding conflicting results. To date, robust data concerning reference values for stimulation tests in healthy adolescents are lacking.

IGF-I and IGFBP-3 may represent helpful markers of GH secretory status (Cianfarani et al., 2005). It has been reported that in transition patients the use of a cut-off value of −2 SDS according to age and sex-related IGF-I levels would miss more than one third of GHD subjects, −2 SDS showing a sensitivity of 62%, −1.7 SDS of 77%, and −1.3 SDS of 87% (Maghnie et al., 2005; Corneli et al., 2007). However, most of the reference values used in these studies was taken from the assay kit sheet without in house validation on a representative control population. Furthermore, it is well known that a remarkable variability among different assay exists due to differences in antibody specificity and/or pre-analytical sample preparation strategies to remove binding protein interferences. This inter-assay variability affects assay standardization (Clemmons, 2011). Recently, high resolution mass spectrometry has been validated for quantitative analysis of IGF-I. This approach offers the advantage of being carried out under conditions that can be tuned to preserve or eliminate biologically relevant interactions (Bystrom et al., 2012). The publication of IGF-I reference values up to the age of 18 years opens avenue for the correct use of IGF-I measurement (Brabant et al., 2003; Elmlinger et al., 2004; Bedogni et al., 2012).

Although IGFBP-3 measurement shows a high specificity in diagnosing GHD (about 100%), sensitivity is poor (about 30%), due to a number of pitfalls which limit reliability and usefulness in clinical practice (Cianfarani et al., 2005).

Magnetic resonance imaging (MRI) is another helpful tool to establish the diagnosis of permanent GHD. It is however worth remembering that not all neuroradiological abnormalities detected at the time of initial diagnosis are indicative of a permanent GH deficiency. While hypothalamic-pituitary disconnections are often associated with persistent deficiency, pituitary hypoplasia (Maghnie et al., 1999; Radetti et al., 2007) and ectopic posterior pituitary may be associated with a normalization of GH secretion (Di Iorgi et al., 2007).

## Deciding on the Optimal GH Replacement Dose in Transition: A Leap in the Unknown

Another challenge in the transition phase is to establish the optimal dose of GH for achieving normal adult height and optimal metabolic profile (Clayton et al., 2005). The usual GH replacement dose for children (25–35 μg/kg/day) and adults (100–300 μg/day) seems in fact inappropriate in transition (Growth Hormone Research Society, 2000; Molitch et al., 2006). A dose of 200–500 μg/day, with the higher doses in girls on estrogen replacement therapy, has been suggested (Clayton et al., 2005). Thereafter, the dosage may be progressively increased up to 400–500 μg/day. Treatment should be tailored to meet the individual requirements on the basis of the clinical response and serum IGF-I concentrations, which should be kept between 0 and +2 SDS (Clayton et al., 2005). A good clinical response should induce to maintain the same GH dose even in presence of sub-optimal IGF-I levels (Clayton et al., 2005).

## Conclusion: Does Transition Really Exist?

Due to the existing conflicting data on both diagnosis and treatment of GHD patients during transition, it has to be pointed out that many of the current approaches are based on arbitrary assumptions rather than on evidence-based medicine. The same definition of transition leaves room to ambiguity. There is no biological basis for considering GHD individuals during a so wide time span under the same label. The different individuals enter puberty at different times; all the more GHD patients have different rates of biological and psychological maturation. Stimulation test cut-offs, GH doses, and responses to GH therapy during transition may be influenced by age, time elapsed from puberty, and underlying diagnoses. It is surprising that in an epoch of individualized medicine and pharmacogenomics, a so simplistic and all-inclusive definition is being still used. Moreover, do we really need that? The diagnosis of permanent GHD should be based on a comprehensive clinical, anthropometric, biochemical, endocrine, and neuroradiological approach rather than arbitrary cut-off laboratory values. Finally, GH dose should be tailored to meet individual requirements for optimizing growth, body composition, bone mineralization, and metabolic homeostasis.

## Conflict of Interest Statement

The authors declare that the research was conducted in the absence of any commercial or financial relationships that could be construed as a potential conflict of interest.
